# Evolution of antibody landscape and viral envelope escape in an HIV-1 CRF02_AG infected patient with 4E10-like antibodies

**DOI:** 10.1186/1742-4690-6-113

**Published:** 2009-12-14

**Authors:** Tessa Dieltjens, Leo Heyndrickx, Betty Willems, Elin Gray, Lies Van Nieuwenhove, Katrijn Grupping, Guido Vanham, Wouter Janssens

**Affiliations:** 1 Department of Microbiology, Unit of Virology, Institute of Tropical Medicine, Antwerp, Belgium; 2 National Institute for Communicable Diseases, Johannesburg, South Africa; 3 Department of Parasitology, Unit of Parasite Diagnostics, Institute of Tropical Medicine, Antwerp, Belgium; 4 Department of Biomedical Sciences, University of Antwerp, Antwerp and Faculty of Medicine and Pharmacy, Free University of Brussels, Belgium

## Abstract

**Background:**

A minority of HIV-1 infected individuals develop broad cross-neutralizing (BCN) plasma antibodies that are capable of neutralizing a spectrum of virus variants belonging to different HIV-1 clades. The aim of this study was to identify the targeted epitopes of an individual with BCN plasma antibodies, referred to as ITM4, using peptide phage display. This study also aimed to use the selected mimotopes as tools to unravel the evolution of the antibody landscape and the viral envelope escape which may provide us with new insights for vaccine design.

**Results:**

This study led us to identify ITM4 plasma antibodies directed to the 4E10 epitope located in the gp41 membrane-proximal external region (MPER). Analysis of antibody specificities revealed unusual immunogenic properties of the ITM4 viral envelope, as not only the V3 loop and the gp41 MPER but also the C1 and lentivirus lytic peptide 2 (LLP2) region seem to be targets of the immune system. The 4E10-like antibodies are consistently elicited during the 6-year follow up period. HIV-1 ITM4 pseudoviruses showed an increasing resistance over time to MPER monoclonal antibodies 4E10 and 2F5, although no changes are found in the critical positions of the epitope. Neutralization of COT6.15 (subtype C; 4E10-sensitive) pseudoviruses with alanine substitutions in the MPER region indicated an overlapping specificity of the 4E10 monoclonal antibody and the ITM4 follow up plasma. Moreover the 4E10-like antibodies of ITM4 contribute to the BCN capacity of the plasma.

**Conclusions:**

Using ITM4 BCN plasma and peptide phage display technology, we have identified a patient with 4E10-like BCN antibodies. Our results indicate that the elicited 4E10-like antibodies play a role in virus neutralization. The viral RNA was isolated at different time points and the ITM4 envelope sequence analysis of both early (4E10-sensitive) and late (4E10-resistant) viruses suggest that other regions in the envelope, outside the MPER region, contribute to the accessibility and sensitivity of the 4E10 epitope. Including ITM4 specific HIV-1 Env properties in vaccine strategies may be a promising approach.

## Background

During the course of Human Immunodeficiency Virus 1 (HIV-1) infection, a huge variety of HIV-1 variants, termed 'quasispecies' are generated. This is driven by a high mutation rate and a high turnover rate of HIV-1 *in vivo*, as well as by selective immune responses. In response to the high degree of antigenic polymorphism, HIV-1 infected patients develop a strong and persistent immune response characterized by CD8^+ ^cytotoxic T-lymphocyte activity and the production of HIV-1 specific antibodies. Antibodies with neutralizing capacities against primary isolates emerge after seroconversion relatively late, and their neutralization spectrum broadens over time [1,2]. Broad cross neutralizing (BCN) antibodies that target conserved regions on diverse HIV-1 clades are generated in a minority of the infected patients during natural infection. Nevertheless some BCN monoclonal antibodies that neutralize HIV-1 *in vitro *have been identified and include IgG b12 (directed against the CD4 binding site), 2G12 (anti-gp120 carbohydrate), 2F5 (anti-gp41) and 4E10 (anti-gp41). Out of this small panel of BCN monoclonal antibodies, 4E10 has the most broadly neutralizing activity described to date [3]. Studies applying passive immunization with these monoclonal antibodies show protection against *in vivo *challenges with SHIV in rhesus macaques [4-7]. In humans, the passive transfer of neutralizing monoclonal antibodies 2G12, 2F5, and 4E10 resulted in a delay of HIV-1 rebound after cessation of antiretroviral therapy [8]. As such, it is hoped that by inducing a sufficiently high BCN antibody concentration in addition to antiviral CD8^+ ^lymphocyte immunologic responses through vaccination, an individual might be protected against HIV-1 by any natural transmission route. One of the current challenges remains to generate immunogens that are capable of inducing a high titer of neutralizing antibodies. However, using envelope (Env) proteins presenting these neutralizing epitopes has not yet resulted in eliciting BCN antibody responses as measured by commonly used neutralization assays [9,10]. The optimal presentation of the corresponding neutralization epitopes may be restricted to the conformational Env context of particular virus variants that induce these antibodies in natural infection [11-14].

In the present study we aimed at unravelling the antigenic landscape of the HIV-1 Env of ITM4, a CRF02_AG infected patient with BCN antibodies, using M13 phage display peptide libraries. Peptide phage display is a simple methodology for screening interactions between antibodies and their epitopes with the major advantage that both linear and conformational B-cell epitopes can be identified without pre-existing notions about the nature of the interaction. Previously, several groups used this technology to successfully map the epitope specificities of serum neutralizing antibodies of HIV-infected individuals We subsequently tested several mimotopes as immunogens [15-17]. We explored the targets of neutralizing antibodies present in the patient's plasma and investigated the evolution of the humoral immune responses during disease progression. The results of the panning revealed the presence of 4E10-like antibodies. Studies by Yuste *et al. *[18] suggest that 4E10 and 2F5-like neutralizing specificities are rare in HIV-1 infected individuals. Furthermore, the initial isolation of the monoclonal antibodies (Mabs) 2F5 and 4E10 was done without reference to the original blood donors; so the viruses of the respective donors have never been isolated and identified. Therefore, studies analyzing the virus envelope evolution in patients with 2F5 or 4E10-like antibodies are of great interest. Recently, an HIV-1 infected patient with 2F5-like antibodies was discovered and analyzed in detail by Shen *et al*. [19]. In addition to this finding, we report here on a patient with 4E10-like antibodies, which we refer to as ITM4. We describe four functional envelope clones isolated at different time points during disease progression. The correlation between viral escape and the presence or appearance of several antibodies was explored. The contribution of the 4E10-like antibody in the broad cross neutralizing activity of the plasma was further examined.

## Results

### Identification of patient ITM4

**Figure 1:**
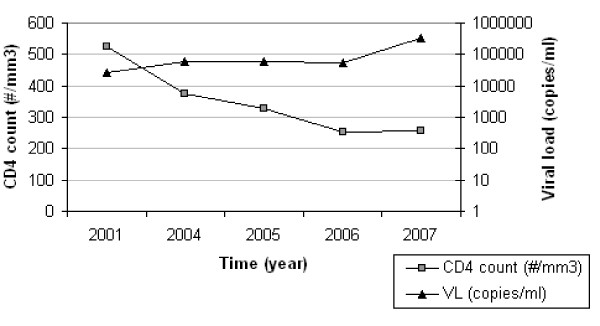
**CD4+ T-cell numbers and viral loads detected in follow up plasma samples of ITM4 over a period of 6 years**.

ITM4 is a male HIV-1 Circulating Recombinant Form CRF02_AG infected individual, who has been infected by heterosexual transmission. He first consulted the Institute of Tropical Medicine in 2001. Between 2001 and 2007, his viremia increased from 42,000 copies/ml (sample ITM4_01.1) to 330,000 copies/ml (sample ITM4_07.2), and his CD4 T cell counts decreased from 550 per mm^3 ^(sample ITM4_01.1) to 250 per mm^3 ^(sample ITM4_07.2) (Fig. 1). During this follow up period, patient ITM4 never received anti-retroviral therapy. ITM4 was selected for the unique capacity of his plasma, taken in 2005 (further referred to as ITM4_05) to neutralize a broad spectrum of primary virus isolates from subtypes A (3/4), B (2/4), C (4/4), D (4/4), CRF01_AE (4/4) and CRF02_AG (5/5) in a primary virus/PBMC neutralization assay (Table 1)[20]. The BCN capacity of his plasma was confirmed for a sample taken in 2007 in a pseudovirus/TZM-bl assay (Table 1). The panel of pseudoviruses tested was neutralized with ID50s ranging between 33 and >640, with the subtypes C and D Envs being the most sensitive and subtypes B Envs being more neutralization resistant.

**Table 1:** Neutralization profile of patient ITM4 in a primary virus/PBMC assay (left) and a pseudovirus/TZM-bl assay (right)

	**PBMC/Primary Virus Assay**	**Plasma Sample ITM4_05**	**Pseudovirus/TZM bl Assay**	**Plasma Sample ITM4_07**
**Subtype**	**Virus**	**% Neutralization^a^**	**Pseudovirus**	**ID50^b^**
		
A	VI 191	66	PV 92RW009	**222**
	92UG037	**100**	PV PIC 32281	54
	VI 820	**99**		
	VI 1031	**100**		
		
B	89.6	0	PV SF162	**322**
	93US076	69	PV JRFL	**88**
	93US077	**97**	PV AC10	**62**
	93US143	**100**	PV CAAN	33
		
C	VI 829	**80**	PV VI 829	**180**
	VI 882	**99**	PV VI 882	**316**
	VI 1358	**99**	PV VI 1358	**208**
	VI 1144	**100**	PV 92BR025	**155**
			PV Du174	**333**
		
D	VI 656	**97**	PV UG024	**>640**
	VI 693	**100**		
	VI 824	**91**		
	VI 865	**93**		
		
CRF01	VI 1249	**98**	PV CM244	**93**
	CA 10	**97**		
	VI 1888	**96**		
	92TH022	**99**		
		
CRF02	VI 1090	**94**	PV VI 1090	**221**
	CA18	**88**	PV CA18	**63**
	VI 2680	**99**		
	VI 1380	**98**		
	VI 2727	**93**		

^a ^% neutralization obtained with 1:20 plasma dilution, ≥80% reduction in virus titer is indicated in bold.

^b ^Plasma dilution causing 50% reduction of relative light units compared to the virus control, ID50 ≥50 is indicated in bold.

### Peptide phage selection and localization

**Figure 2:**
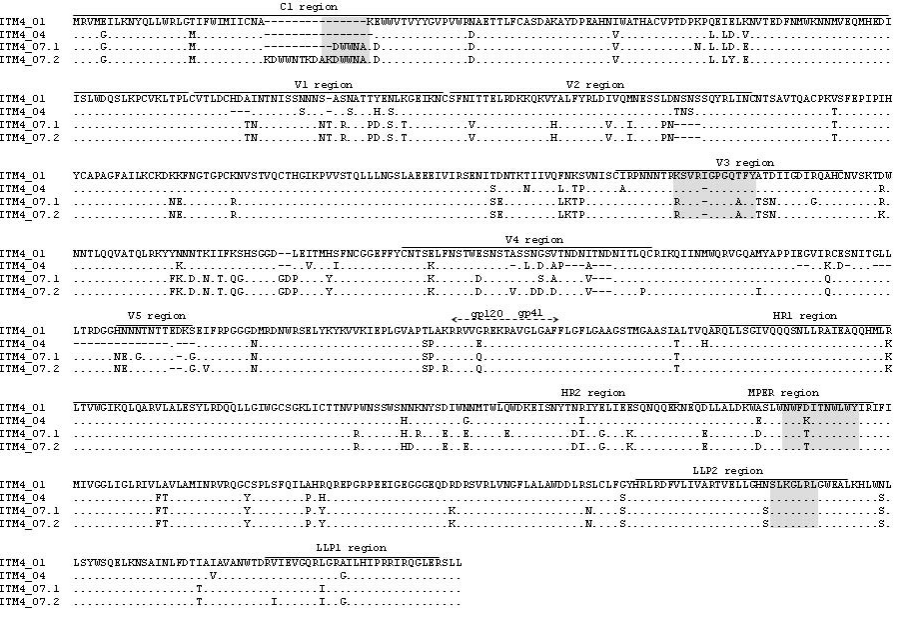
**Env amino acid alignment of ITM4 follow-up pseudoviruses**. Dots are included for alignment purposes. Mimotope localizations are highlighted in grey.

In order to map the antibody responses directed against the HIV-1 envelop in patient ITM4, peptide phage display technology was applied. A random 12-mer phage library was panned against a pool of ITM4 plasma samples. After 3 selection rounds, peptide sequences were deduced for phage displaying positive reactivity in ELISA with ITM4 plasma, as well as low or no reactivity with an HIV negative plasma pool. The generated peptide sequences were aligned and ranked according to homology, resulting in four groups of peptide sequences with a commonly similar motif (Table 2). Phage clones presenting a peptide with a NWFNLTQTLMPR motif were predominantly documented (n = 18); twelve peptide phages represented the KxWWxA motif. Furthermore, mimotopes with a SLxxLRL motif (n = 7) and a KxxxIGPHxxY motif (n = 3) were identified. Peptide sequences were compared with the linear Env sequences of ITM4 to localize each of the mimotope groups. The NWFNLTQTLMPR peptide shares key amino acid residues of the 4E10 epitope [WFx(I/L)(T/S)xx(L/I)W] located in the membrane-proximal external region (MPER) of gp41 [21,22]. Mimotopes with the KxxxIGPHxxY motif showed homology to the crown of the V3 loop of the ITM4 gp160 sequences (Table 2). The KxWWxA motif shared linear homology to C1 sequences of ITM4 isolates of 2007 (Fig. 2). A last group of mimotopes sharing the SLxxLRL motif is predicted to bind antibodies directed to the lentivirus lytic peptide 2 (LLP2) region of gp41.

**Table 2:** Overview of the selected mimotope groups

Mimotope AA Motif	Location in the ITM4 Env Sequence	Times Selected	Cross Reactivity ^a^
NWFNLTQTLMPR	gp41 MPER region	18	1/80 (1.3%)
KxWWxA	gp120 C1 region	12	0/80 (0.0%)
SLxxLRL	gp41 LLP2 region	7	1/80 (1.3%)
KxxxIGPHxxY	gp120 V3 region	3	10/80 (12.5%)

^a^ Number of HIV-1^+^ plasma samples cross-reacting with the mimotope group.

### Recognition of the ITM4 phage mimotopes by other HIV-1 infected individuals

In a capture ELISA, eighty random plasma samples of HIV-1 positive individuals were screened for antibody cross reactivity to the selected ITM4 peptide phage groups. The highest cross-reactivity was seen for the mimotope representing the immunodominant part of the V3 region, ten (12.5%) of the tested HIV1 plasma had antibodies that bound this mimotope (Table 2). The mimotope localized in the gp41 MPER is more exclusive; it was only recognized by one plasma sample (later referred as CrossR1). This is in accordance with previous publications indicating that antibodies against the MPER are found in a minority of HIV infected individuals [18,23-25]. The random plasma samples also showed a very weak cross reactivity (1/80) towards the LLP2 mimotope, while none of the plasma samples that were tested recognized the C1 mimotope. This result indicated that this epitope is unique for the virus circulating in ITM4.

### Evolution of the antibody development in ITM4 follow up plasma samples

**Figure 3:**
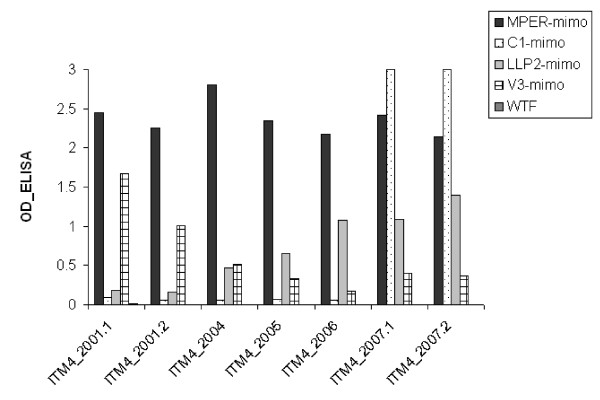
**Reactivity in ELISA of ITM4 follow up plasma samples between 2001 and 2007 with the selected mimotope groups**. (WTF: wild type phage).

Next, an ELISA was performed to determine the reactivity of the different phage groups with the individual plasma follow up samples of ITM4 (2001-2007) (Fig. 3). Reactivity patterns of the peptide phage groups with ITM4 follow up plasma were not uniform. Each phage group had a unique reaction pattern: (1) Antibodies elicited against the MPER (NWFNLTQTLMPR) mimotope were already present in 2001 and showed a comparable high reactivity in all the follow up samples, whereas (2) the antibodies against the C1 (KxWWxA) mimotope were absent in most of the samples and only appeared in the plasma samples taken in 2007. The V3-specific antibodies (3) showed a gradual decreasing reactivity over time; in contrast (4) an increase of binding antibodies is seen for the LLP2 (SLxxLRL) mimotope. These data demonstrate that the antibody development in ITM4 is a continuously dynamic event.

### Specificity of the MPER mimotope

**Figure 4:**
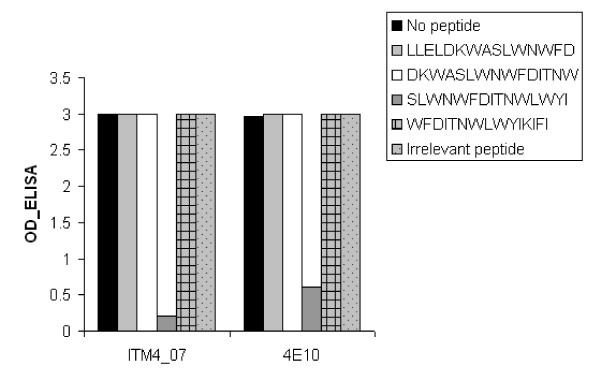
**Competitive ELISA screening peptides for their ability to compete with the 4E10 mimotope in binding to ITM4 plasma and 4E10 Mab**. Overlapping peptides stretching the 2F5 and 4E10 epitopes are used for competition. An irrelevant peptide was included as negative control.

To further explore the characteristics of the antibodies binding to the NWFNLTQTLMPR mimotope, additional ELISA experiments were performed. First, the ability of the monoclonal antibody 4E10 to bind this phage peptide was analyzed. We observed a high signal (OD = 3.0) when 4E10 was added to the mimotope, indicating the ability of the NWFNLTQTLMPR peptide to bind 4E10-like antibodies (Fig. 4). In a second part of the experiment, different peptides overlapping the 4E10 region were used in a competition ELISA. The results clearly demonstrated that peptide 6376 obtained from the NIH AIDS Reagent Program, SLWNWFDITNWLWYI, presenting the 4E10 epitope, strongly competed with the peptide phage for 4E10-binding (Fig. 4). A similar observation was made for the plasma from ITM4 (Fig. 4), the same peptide occupied the binding places for the antibodies binding the NWFNLTQTLMPR mimotope as for the monoclonal antibody 4E10. Taken together, the results above suggest that 4E10-like antibodies are present in our subject of interest.

### Autoreactive antibodies

As shown by Haynes *et al. *[26] and Scherer *et al*. [27], Mab 4E10 has an affinity for the autoantigen cardiolipine (CL), due to the epitope position which is recognized in the context of the viral membrane. In our study, the cross-reactivity to CL of five broadly cross neutralizing plasma samples was analyzed, including both patients with antibodies against the NWFNLTQTLMPR mimotope: ITM4 and CrossR1. The presence of anti-CL antibodies was measured in an ELISA, and plasma samples were ranked according to their reactivity, with >20 GPL units categorized as positive; 10-20 GPL units as weakly positive, and < 10 GPL units as negative. Plasma ITM4_07 showed the highest reactivity (34 GPL units) and thus scored strongly positive. The second highest score (16 GPL units) was obtained with plasma from patient CrossR1. Two other BCN plasma were weakly positive (12 and 11 GPL units respectively), one BCN plasma scored negative (8 GPL units) (data not shown). Additionally, we analyzed the presence of other auto-antibodies (anti dsDNA, anti Ro/Ssa, and anti Jo1 antibodies) in the selected plasma samples. None of the samples showed positive reactivity with any of these auto-antigens (data not shown).

We further noted that the clinical status of patient ITM4 was regularly followed between 2001 and 2007 by the team of medical doctors at the clinic of the Institute of Tropical Medicine (Antwerp, Belgium). The fact that no symptoms of auto-immune disease were reported in this follow up period, suggests that the ITM4 antibodies reacting with the CL autoantigen are non-pathogenic, induced by the HIV-infection, and are not present due to an autoimmune disorder [28].

### Genotypic Analysis of the MPER of ITM4

**Figure 5:**
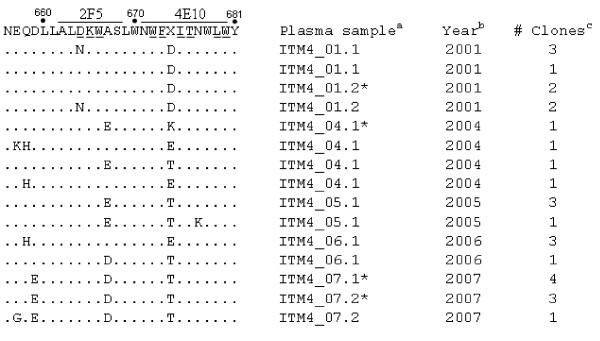
**Sequence characteristics of the gp41 MPER of ITM4 viruses isolated at different time points**. The consensus MPER sequence is designated in the first line. The core epitopes of 2F5 and 4E10 are indicated, the key amino acid residues of both epitopes are underlined. MPER sequences derived from a functional Env clone are marked by an asterisk. ^a ^Plasma samples used for viral RNA isolation. ^b^Year of sampling. ^c ^Number of clones having this motif.

MPER sequences of 4 clones per follow-up sample (n = 7) were generated and analyzed (Fig. 5). For clones of samples ITM4_01.1 and ITM4_01.2 taken in 2001, two diverse 2F5 epitope variants were documented: ALDKWA and ALNKWA, having a D664N substitution. Clones of samples from 2004 and later time points displayed wild type ALDKWA and/or A667 mutant sequences (Fig. 5). ALDKWA represents the consensus subtype A 2F5 epitope, whereby the DKW motif is crucial for binding 2F5 [29,30]. The D664N substitution resulting in ALNKWA, has been described as a 2F5 escape variant with a lower but still relatively high infectivity *in vitro *and displaying resistance to 2F5 neutralization [31].

The 4E10 epitope of clones of ITM4 follow-up samples did not present mutations in key amino acids [WFx(I/L)(T/S)xx(L/I)W]. The subtype A 4E10 epitope NWFDITNWLW was conserved in clones of samples ITM4_01.1 and ITM4_01.2 taken in 2001. Clones of 2004 and samples of later time points displayed D674 mutant sequences. One N677K mutation is seen in a clone isolated from the 2005 sample. Both mutations at position 674 and 677 do not confer resistance to 4E10 [21], but substitutions at these positions may have an impact on 4E10 neutralization sensitivity [32].

### Neutralization sensitivity of functional ITM4 clones

To investigate the autologous neutralizing activity, we cloned and pseudotyped full length envelope genes of functional clones from 4 different time points (2001, 2004, 2007.1, 2007.2) and examined the sensitivity of the variants to autologous plasma of the same time points in a pseudovirus/TZM-bl assay. At all of the time points analyzed, the titers against earlier virus were higher than against contemporaneous or later virus, suggesting a constant change in immune pressure and viral escape (Table 3). The highest neutralizing activity was seen against the earliest pseudovirus (PV ITM4_01), lower neutralization sensitivity was observed for the clones of later time points (PV ITM4_07.1 and PV ITM4_07.2).

**Table 3:** Susceptibility of ITM4 pseudoviruses isolated at different time points to neutralization by autologous plasma and by Mabs.

**Neutralizing activity of **	**Autologous follow up plasma samples (ID50)^a^**					**Monoclonal Antibodies (IC50)^b^**			
	**ITM4_2001**	**ITM4_2004**	**ITM4_2005**	**ITM4_2007.1**	**ITM4_2007.2**	**MAb 4E10**	**MAb 2F5**	**MAb 2G12**	**MAb b12**
		
**Pseudoviruses (PV)**									
PV ITM4_01	540	1905	>4680	4650	4475	**0.23**	**0.21**	>25	>25
PV ITM4_04	118	194	255	219	216	**11.5**	**19**	>25	>25
PV ITM4_07.1	<25	<25	<25	111	33	**17.4**	**>25**	12.75	>25
PV ITM4_07.2	<25	<25	<25	67	33	**>25**	**>25**	>25	>25

^a^Plasma dilution causing 50% reduction of relative light units compared to the virus control.

^b ^Antibody concentration (μg/ml) causing 50% reduction of relative light units compared to the virus control.

Cross neutralizing monoclonal antibodies 4E10, 2F5, 2G12 and b12 were used to identify the neutralization sensitivity patterns of the four different ITM4 pseudoviruses. Large variation in neutralization susceptibility of the pseudoviruses was seen, as shown in Table 3. The earliest virus (PV ITM4_01), isolated in 2001, was very sensitive to both 4E10 and 2F5, with IC50s at concentrations 0.23 and 0.21 μg/ml respectively. The isolate from 2004 (PV ITM4_04), showed already a 50-fold decrease in sensitivity to 4E10 and a 90-fold decrease in sensitivity to 2F5. The virus isolates from 2007 (PV ITM4_07.1 and PV ITM4_07.2) showed complete resistance to 2F5 (>25 μg/ml) and demonstrated also very low (17.4 μg/ml; PV ITM4_07.1) or no (>25 μg/ml; PV ITM4_07.2) sensitivity to 4E10. Notably, the emerging virus was able to create resistance to 2F5 and 4E10 during disease progression. No neutralization of any of the pseudoviruses was observed by Mab b12 (25 μg/ml), and only one of the four isolates (PV ITM4_04) showed moderate susceptibility to 2G12 (with an IC50 at 12.75 μg/ml).

### Evolution of the viral envelope

Sequence variability over time in the HIV-1 envelope of ITM4 was examined in the infectious pseudotyped viruses; the functional clones were sequenced and aligned (Fig. 2). To determine whether the evolution of Env correlated with the development of HIV-1 antibodies in ITM4, we analyzed the target regions of the ITM4 antibodies. The V3 region of the generated variants shows only a small amount of variation. The decrease in titer of V3 antibodies over time in patient ITM4 (Fig. 3) can therefore not be explained by mutations or deletions or insertions in the V3 sequence respectively. One suggestion could be that other regions in gp120 influence the presentation and accessibility of the V3 loop [33]. The second region of interest is the first constant region (C1) of gp120, an amino acid sequence AKxWWx present in ITM4_01 was duplicated in ITM4_07.1 and triplicated in ITM4_07.2. This multiplication event in the C1 region is very unusual and unique for the virus circulating in ITM4, as none of the Env references available from the HIV Database http://www.hiv.lanl.gov show a similar insertion. The expansion of the Env C1 region as a result of the insertion of the AKxWWx sequence seems to stimulate the immune system to produce antibodies against this sequence. As shown in Figure 3, high titers of antibodies were found in both 2007 plasma samples directed against the KxWWxA mimotope. A third target region of the anti-HIV envelope antibodies is the LLP2 region in gp41. The presence of antibodies directed against this part of gp41 supports the possibility that the LLP2 domain is (transiently) exposed. Antibody titers in the follow up plasma samples suggest an enhanced exposure in clones at later time points. Only one amino acid substitution is seen in the LLP2 domain, a hydrophilic asparagine (N) at position R788 left of the SLxxLRL mimotope, is changed to a serine (S) in the isolates from 2007. The last target of the ITM4 antibodies is part of the MPER of gp41. In none of the clones sequenced was significant substitutions found in this region, as discussed above, despite the high antibody titers found in all the follow up plasma samples.

### Contribution of 4E10-like antibodies to neutralization

We next tested if the ITM4 antibodies sharing the 4E10 epitope may contribute to or be responsible for the BCN capacity of ITM4. For this purpose, a panel of mutant viruses (COT6.15 mutants) with substitutions of charged amino acids for alanine residues at positions 667 to 680 in the MPER of gp41 was used. The variation in neutralization sensitivity of wild type COT6.15 virus and mutants was measured in a pseudovirus/TZM-bl assay. We analyzed changes in IC50 values of two ITM4 plasma samples (ITM4_01 and ITM4_07). The CrossR1 sample and Mab 4E10 were included as controls. Results from these experiments are shown in Table 4. For all samples tested, an increased neutralization resistance is observed when residues were replaced at positions N668A, F673A and D674A. Substitution at position T676A decreased the sensitivity of all the samples except for ITM4_07.

**Table 4:** The effect of charged residue-to-alanine replacement on neutralization capacity of ITM4 plasma, CrossR1 plasma and Mab 4E10

VIRUS	COT6.15	MAB 4E10	ITM_01	ITM4_07	CROSSR1
**AA**	**MUTATION**	**IC50^a^**	**IC50^b^**	**IC50^c^**	**IC50^d^**

	WT	1,11	603	3544	249
K	667A	0,43	556	>4860	367
**N**	**668A**	**4,63**	**117**	**472**	**50**
L	669A	0,15	929	>4860	528
W	670A	0,93	445	>4860	163
S	671A	0,23	644	>4860	211
**W**	**672A**	**>25**	241	4189	115
**F**	**673A**	**>25**	**70**	**1177**	**39**
**D**	**674A**	**4,43**	**63**	**516**	**29**
I	675A	0,15	333	>4860	193
**T**	**676A**	**>25**	**188**	2069	**61**
K	677A	0,38	640	4285	180
W	678A	0,22	801	>4860	391
L	679A	0,49	317	>4860	398
**W**	**680A**	**4,00**	479	>4860	360

AA: amino acids in the 4E10 region of the COT6.15 virus

Alanine-replacements inducing a 3-fold or higher decrease in sensitivity are indicated in grey.

^a^Antibody concentration (μg/ml) causing 50% reduction of relative light units compared to the virus control.

^b ^Plasma dilution causing 50% reduction of relative light units compared to the virus control.

Substitutions at positions W672A and W680A, seem to only have a significant impact on the IC50 for the control Mab 4E10. The IC50 values were not changed dramatically by the respective charged residue-to-alanine replacement at the other positions (667, 669, 670, 671, 675, 677, 678 and 679). Overall, our results indicate the presence of 4E10-like antibodies in both ITM4 plasma and CrossR1 plasma of which the epitope overlaps with the Mab 4E10 epitope in key positions for neutralization. The decrease in neutralization capacity of ITM4 by the introduced substitutions in the region overlapping the 4E10 epitope confirmed that the 4E10-like antibodies present in the plasma samples of 2001 and 2007 do contribute to the neutralization breadth in this patient.

## Discussion

The search for a prophylactic HIV vaccine is focused on the discovery of novel antibody specificities and their associated viral epitopes that could be useful for immunogen design. Recently, several groups studied the specificities of BCN antibodies and revealed that antibodies directed to the gp120 CD4 binding site and the gp41 MPER contribute to the exceptional neutralizing capacity of BCN plasma samples [23-25,34]. Besides this, they noted that a major part of the neutralizing activity still remains undefined, and therefore efforts to map additional neutralizing epitopes may be useful for the development of an HIV vaccine. In this study we aimed to identify key epitopes of HIV-1 Env involved in the broadly cross neutralizing capacity of patient ITM4. Plasma of this subtype CRF02_AG infected individual was screened using M13 peptide phage display libraries in order to identify the epitopes that are potentially involved in the generation of ITM4's neutralizing responses against a wide variety of HIV-1 strains. In order to select peptide phage corresponding to linear and conformational Env epitopes, potentially binding neutralizing antibodies, we adopted a strategy of positive and negative selections. The mimotope sequences of the ITM4 specific peptide phage were determined and localized in the gp160 sequence. Four groups of mimotopes were identified, the so called MPER mimotopes, the V3 mimotopes, the C1 mimotopes and the LLP2 mimotopes, indicating that phage libraries can be applied to identify various Env epitopes, as previously published [16,17,35]. Evaluation of peptide phage for antibody binding in ITM4 follow-up samples revealed a different pattern for each of the peptide phage, illustrating the dynamic process between immune system and virus. Of interest, the only region which is immunogenic over the complete follow up period is the MPER epitope. Antibodies against the AKxWWN epitope in the C1 region only appear after a multiplication of this sequence. The V3 epitope seems to be less accessible on the later viruses. In contrast, the LLP2 epitope is more exposed on the later viruses than on the earlier viruses. A study by Lu *et al*. showed that the LLP2 region, which is part of the cytoplasmic tail of gp41, is transiently exposed during the fusion process of the virus with the target cell [36]. A slower fusion process could cause the appearance of antibodies against this region. Variation in time may contribute to the escape from antibody pressure directed to the Env receptor domains by changing the exposure of neutralization sensitive epitopes [37-39].

As the MPER is known to be an interesting target for vaccine design, we focused our experiments on the MPER antibodies present in the studied patient. The identification of naturally induced 4E10-like antibodies is of major importance as 4E10 binds and neutralizes virtually all HIV-1 viruses regardless of their subtype [3]. Besides, the 4E10 epitope is conserved in all HIV-1 viruses and thus is crucial for infection. Our observations support results made by others showing that antibodies against the 4E10 epitope are rarely encountered in HIV-1 positive individuals [18,23-25]. Only one of the eighty HIV-1^+ ^plasma tested in our binding study cross-reacted with the MPER mimotope. A detailed study of both ITM4's viral envelope and antibody landscape could provide crucial information on how to present the epitope in an ideal way to the immune system to induce potent neutralizing antibodies since the Env of the ITM4 virus may have adapted a conformation whereby the 4E10 epitope is exposed to the immune system.

Firstly, the specificity of the antibody binding the MPER-mimotope was characterized in a competition assay by screening overlapping peptides that map the 2F5 and 4E10 epitope. This test proved that both the ITM4 MPER-directed antibodies and the Mab 4E10 had a binding affinity for the same epitope. Secondly, as the 4E10 epitope is located close to the viral membrane, the monoclonal antibody 4E10 is shown to both bind lipids from the membrane as well as the peptide epitope located on the envelope [40]. As a consequence of this affinity for the lipid membrane, the antibody was previously described as autoreactive, binding the autoantigen cardiolipin [26,27]. The ELISA results indicated high cross-reactivity with cardiolipin in the ITM4 plasma sample. This high cross-reactivity supports our presumption that 4E10-like antibodies are circulating in the patient.

In a next phase, we took a closer look at the viral envelope of ITM4. The neutralization profiles were determined using pseudotyped viruses expressing Envs of different time points. The autologous neutralization data of patient ITM4 suggest a continuous escape of the virus from antibody pressure over six years. The evolving humoral immune response is rather high in potency against the earliest autologous virus. The latest virus exhibited very low sensitivity to the latest plasma samples and resistance to the earlier autologous plasma samples, which can be due to a continuing evolution of the viral envelope sequence. The neutralization experiments with the MPER monoclonal antibodies 2F5 and 4E10 revealed an interesting phenomenon. The earliest virus seems to be very sensitive to both monoclonal antibodies, suggesting that this region is highly accessible on the viral envelope. However, a decreased sensitivity to neutralization by 2F5 and 4E10 was seen for the second pseudovirus, isolated 3 years after the first one was found. The most recent viral envelope, isolated 6 years after the first one, is phenotypically resistant to both MPER Mabs 2F5 and 4E10, indicating that neutralization escape mutants had emerged and that the 4E10-like antibodies are exerting pressure on viral replication. In order to escape from antibody pressure a virus can change the epitope specifically targeted by the antibody or influence the presentation of the epitope by changing the structural context through mutations in other regions. Specifically, the accessibility of the gp41 epitopes to neutralizing antibodies may be sterically blocked by the folding of the variable loops of gp120 and/or the glycan shield in gp120 or other regions of gp41 [41]. A study by Zwick *et al *[30] revealed the amino acid positions in the MPER which cause resistance to 2F5 and 4E10 neutralization by inducing alanine substitutions in both epitopes. For 2F5, the positions D664, K665 and W666 play a major role in the binding and recognition of the epitope. In the case of 4E10, resistance occurred by substitutions at position W672, F673 and W680. Our analysis of the MPER of the infectious ITM4-pseudoviruses could not correlate phenotypical resistance to both Mabs 4E10 and 2F5 with changes in the critical amino acid positions of their epitopes. In another study by Gray *et al. *[32], 4E10 resistant escape mutants were described; those authors identified some additional positions which may influence the presentation of the 4E10 epitope and thereby change the sensitivity of the viral envelope to 4E10 neutralization. In the latter study, both N674 and N677 had an effect on the sensitivity to 4E10, and we observed a amino acid change at position N677 at different time points. Moreover it was shown that changes in the LLP2 region also could interfere with the sensitivity to Mab 4E10 [32]. This raised the possibility that other regions outside the MPER may be responsible also for the occurrence of resistance against the MPER Mabs. It is not clear if the mutation of the ITM4 virus in the LLP2 region on its own, or in combination with N677, affects the 4E10 sensitivity; nevertheless simultaneously with the occurrence of 4E10 resistance, the antigenicity of the LLP2 region increases, suggesting a change in the envelope structure. Another remarkable change in the gp160 sequence during Env evolution is the unique insertion in the C1 region. The C1 region is part of the inner domain of the gp120 core and interacts with gp41, contributing to the non-covalent binding of gp41 and gp120. Mutagenic studies showed the important role of this region on viral entry [42]. Together with the fact that the C1 region is known to be less variable [43], the C1 epitopes are interesting targets for neutralizing antibodies. Previously, a report by Sreepian *et al*, described the presence of antibodies against the C1 region in a subtype CRF01_AE infected HIV-1 individual, however, immunization studies performed with C1 epitopes did not result in neutralizing antibodies [44,45]. The role of the C1 antibodies in the neutralization capacity of ITM4 should be further analyzed. Moreover, the effect of the duplication event in the C1 region should be examined to reveal its role in the occurrence of 4E10 resistance.

To show the contribution of the 4E10-like antibodies to the neutralizing capacity of the ITM4 plasma, we used a 4E10 sensitive subtype C virus (COT6.15) with alanine-replacements at several positions in the 4E10 epitope. The results confirmed that the neutralization capacity of the ITM4 plasma is partially due to the presence of 4E10-like antibodies. This is consistent with the findings of Gray *et al.*, showing that anti-MPER antibodies are responsible for BCN activity found in some plasma [46]. Furthermore, a major overlap was seen between the residues affecting 4E10 neutralization and ITM4 neutralization, indicating the similar specificities of both antibodies.

## Conclusions

In summary, the conserved 4E10 epitope is highly targeted by vaccine developers, but none has succeeded to generate an antigen capable of eliciting 4E10-like antibodies. Here we provide data that the 4E10 region is not only accessible in patient ITM4, but also immunogenic. ITM4 Env sequence analysis indicates unique gp120 C1 insertions that may have an impact on gp41 conformation and 4E10 epitope presentation. Furthermore, this case confirms that 4E10-like antibodies with neutralizing characteristics can be elicited during HIV infection, and thus the inclusion of ITM4 envelope properties in a prophylactic vaccine might be very promising. However, researchers should take into account that once infection has occurred, neutralizing antibodies can easily be evaded by escape mutants, resulting in "normal" disease progression, as shown in this patient.

## Methods

### Human plasma, Antibodies and Peptides

Plasma samples were obtained from HIV-1 seropositive individuals attending the clinic at the Institute of Tropical Medicine, Antwerp (ITM). All samples were heated at 56°C for 30 minutes to inactivate complement. The studies have been approved by the ITM Institutional Review Board. HIV-1 Mabs 2F5, 4E10, 2G12 and b12 were purchased from Polymun Scientific (Vienna, Austria). Peptides were obtained through the AIDS Research and Reference Reagent Program, Division of AIDS, NIAID, NIH.

### Peptide Phage Display

A New England Biolabs Ph.D.-12 Phage Display Peptide Library Kit (Westburg BV, Leusden, Belgium) was panned for selection of peptide phage binding IgG from a pool of nine ITM4 plasma samples collected between 2001 and 2007, as described previously [16]. Briefly, plasma IgG were linked to magnetic microbeads (Dynabeads M-450 Tosylactivated; Invitrogen, Merelbeke, Belgium) coated with an anti-human IgG (Fc-specific; Lucron bioproducts, De Pinte, Belgium). Peptide phage were selected from the library of >2 × 10^9 ^random peptides by performing alternately positive (~pool of ITM4 IgG) and negative (~pool of HIV negative IgG) selection rounds. Panning was repeated 3 times on amplified phage eluate to enrich for peptide phage binding specifically to the target antibodies. Phage collected after the third positive selection round were titrated and single clones were randomly picked and subjected to analysis by capture ELISA and DNA sequencing.

### Antibody binding assay (ELISA)

A capture ELISA was used to identify peptide phage binding to the target antibodies. Microtiter plates were coated overnight at 4°C with a 1/10^4 ^plasma-dilution in phosphate buffered saline (PBS). Plates were blocked for 2 hours with 5% skimmed milk powder in PBS (5%MPBS) at 37°C and washed 3 times with 0.01%Tween-20/PBS. An amount of 10^11 ^phage in 1%MPBS were added and left overnight at 4°C. The plates were washed 4 times before adding HRP-conjugated anti-M13 monoclonal antibody (GE Healthcare, Diegem, Belgium) 1/2000 diluted in 0.01%Tween-20/PBS. After 1 h, color development was performed with ortho-phenylenediamine dissolved in citrate buffer (pH 5) with 0.001% H_2_O_2_. Plates were incubated in the dark at room temperature and results were expressed as difference between OD405nm and OD620nm, read with an automated ELISA reader.

### Competitive ELISA

Competitive ELISA assays were performed as described above, 10 μg/ml peptide was added to the antibody-coated plates 1 h before the phage were applied.

### Sequencing of DNA inserts

Reactive peptide phage were amplified in *E. coli *and single-stranded DNA was isolated using a QIAprep Spin M13 Kit (Qiagen Benelux BV, Venlo, The Netherlands). Sequences encoding the phage peptides were generated, edited, translated and analyzed using Lasergene Software (DNASTAR, Wisconsin, USA).

### Autoreactive antibody assay

The anticardiolipin antibodies were measured in an ELISA assay as previously described [47]. Briefly, wells of polystyrene microtiter plates were coated with 30 μl of CL (from bovine heart, Sigma, St Louis, MO) dissolved in ethanol (50 μg/ml) and evaporated overnight at 4°C. Then, the wells were blocked with 150 μl of a mixture of 1% (w/v) bovine serum albumin (Invitrogen, Merelbeke, Belgium) in PBS for 1.5 hour at room temperature. Next, the wells were washed two times with PBS. Plasma specimens, diluted 1:50 in 1%BSA/PBS, were added (100 μl/well) to the CL-coated wells and incubated for 2.5 h at room temperature. After washing three times with PBS, horseradish peroxidase conjugated goat anti-human IgG (Fc specific) (Biosource, Nivelles, Belgium) was diluted 1:5000 in 1%BSA/PBS, added to the wells (100 μl/well) and incubated for 1.5 hour at room temperature. After washing, ortho-phenylenediamine dissolved in citrate phosphate buffer (pH 5) and urea hydrogen peroxide, were added to the wells (100 μl) to initiate color reaction. The color reaction was stopped by addition of 50 μl/well of 2 M H_2_SO_4_. Optical densities (ODs) were measured at 492 nm using an ELISA reader. Results were expressed in GPL/ml units according to Harris *et al*. [48].

Anti-dsDNA antibodies were detected by the indirect immunofluorescence on Crithidia lucilliae. In brief, Crithidia lucilliae, fixed on wells of a glass slide, was used as a substrate. A series of dilutions (starting at 1:10) of patient plasma was added to the wells. After 30 minutes of incubation the wells were washed three times with phosphate-buffered saline (PBS) and FITC-labelled anti-human IgG (The Binding Site, BMD, Antwerp, Belgium) was added to the wells to induce an antigen-antibody reaction. After washing the wells, the fluorescence of the kinetoplast was evaluated under a fluorescence microscope. A positive test was considered at a titer of 1:10 or above [49].

The anti Ro/SSa and anti Jo1 antibodies were measured in an immunodot (ENA-DOT 7, BMD, Antwerp, Belgium) as written in the manufacturer's instructions.

### HIV-1 viral isolates and pseudoviruses

The origins and sources of the virus isolates have been described previously (Davis *et al.*, 2003) [20]. The following reagents were obtained through the NIH AIDS Research and Reference Reagent Program, Division of AIDS, NIAID, NIH: 89.6 (R. Collman; cat# 1966); 92US076 (J. Sullivan; cat# 2453), 92US077 (J. Sullivan; cat# 2454), 93US143 (M. Robb; cat# 2755); 92TH022 and 92UG037 (The UNAIDS Network for HIV isolation and characterization, and DAIDS, NIAID; cat# 1743 and cat# 1690). The virus Env plasmids 92RW009, SF162, AC10, CAAN, 92BR025, Du174, UG024 and CM244 were obtained from L. Heyndrickx through the NeutNet program [50]. JRFL was kindly provided by D. Burton (San Diego, USA). The virus plasmids for wild type and mutant COT6.15 Envs were kindly provided by E. Gray (Johannesburg, South Africa).

### HIV-1 Env expression plasmid clones

The virus plasmids PIC32281, VI829, VI882, VI1358, VI1090, CA18, ITM4_01, ITM4_04, ITM4_07.1 and ITM4_07.2 were generated from extracted plasma RNA (QiaAmp^® ^Viral RNA Minikit, Qiagen Benelux BV, Venlo, The Netherlands). The complete env sequence was obtained by RT-PCR, followed by nested PCR, and was cloned in an expression vector (pcDNA4/TO; Invitrogen, Merelbeke, Belgium) [51].

### Production of Env-pseudotyped viruses

Cotransfection of 293T cells with an HIV-1 Env expression vector and pNL4-3.Luc. R-E- (obtained from NIH AIDS Research and Reference Reagent Program and contributed by Nathaniel Landau) was evaluated for the generation of luciferase-encoding HIV-1 virions pseudotyped with the desired HIV-1 Env proteins [51].

### Sequence analysis

Env clones of infectious ITM4 pseudoviruses were sequenced. The sequences were assembled and edited using DNASTAR software (Lasergene, Wisconsin, USA). The HIV-1 env nucleotide sequence data were deposited in the EMBL Genbank and DDBJ nucleotide sequence databases under following accession numbers: FN432725-FN432728.

### Pseudovirus neutralization assay

Pseudovirus neutralization assays were conducted using Mabs and autologous patient plasma as previously described [52], http://www.europrise.org/neutnet_sops.html; SOP10. Infection levels were determined after 48 h, by measuring luciferase activity. ID50/IC50 was calculated as the reciprocal plasma dilution antibody concentration (μg/ml) respectively causing 50% reduction of relative light units compared to the virus control.

### Primary virus neutralization assay

The primary virus neutralization assay used, is in detail described on the Europrise website: http://www.europrise.org/neutnet_sops.html; SOP6B. Neutralization ≥80% was considered potent.

## Competing interests

The authors declare that they have no competing interests.

## Authors' contributions

TD performed the majority of the experimental work and the data analysis and drafted the manuscript. LH, BW, LVN and KG contributed to the experimental work. EG contributed to the study design and provided HIV Env plasmids. GV and WJ contributed to the study design and were involved in critically revising the manuscript.
